# Adenoviral L4 33K forms ring-like oligomers and stimulates ATPase activity of IVa2: implications in viral genome packaging

**DOI:** 10.3389/fmicb.2015.00318

**Published:** 2015-04-21

**Authors:** Yadvinder S. Ahi, Sai V. Vemula, Ahmed O. Hassan, Greg Costakes, Cynthia Stauffacher, Suresh K. Mittal

**Affiliations:** ^1^Department of Comparative Pathobiology, College of Veterinary Medicine, Purdue UniversityWest Lafayette, IN, USA; ^2^Purdue University Center for Cancer Research, Purdue UniversityWest Lafayette, IN, USA; ^3^Bindley Bioscience Center, Purdue UniversityWest Lafayette, IN, USA; ^4^Department of Biological Sciences, Purdue UniversityWest Lafayette, IN, USA

**Keywords:** IVa2, 33K, adenovirus, genome packaging, ATPase

## Abstract

The mechanism of genome packaging in adenoviruses (AdVs) is presumed to be similar to that of dsDNA viruses including herpesviruses and dsDNA phages. First, the empty capsids are assembled after which the viral genome is pushed through a unique vertex by a motor which consists of three minimal components: an ATPase, a small terminase and a portal. Various components of this motor exist as ring-like structures forming a central channel through which the DNA travels during packaging. In AdV, the IVa2 protein is believed to function as a packaging ATPase, however, the equivalents of the small terminase and the portal have not been identified in AdVs. IVa2 interacts with another viral protein late region 4 (L4) 33K which is important for genome packaging. Both IVa2 and 33K are expressed at high levels during the late stage of virus infection. The oligomeric state of IVa2 and 33K was analyzed in virus-infected cells, IVa2 and 33K transfected cells, AdV particles, or as recombinant purified proteins. Electron microscopy of the purified proteins showed ring-like oligomers for both proteins which is consistent with their putative roles as a part of the packaging motor. We found that the ATPase activity of IVa2 is stimulated in the presence of 33K and the AdV genome. Our results suggest that the 33K functions analogous to the small terminase proteins and so will be part of the packaging motor complex.

## Introduction

Adenoviruses (AdVs) are non-enveloped icosahedral viruses with a linear dsDNA genome ranging in size from 28 to 45 kbp. The AdV genomes carry conserved AT-rich repeat regions, named ‘A-repeats,’ located between the left inverted terminal repeat (ITR) and the transcriptional start site of early region (E) 1 (E1) in the viral genome (Kosturko et al., 1982; Ostapchuk and Hearing, 2003a,b, 2005; Xing and Tikoo, 2003, 2007). This set of A-repeats constitutes the packaging domain (PD) of an AdV genome. The PD of the human AdV serotype 5 (HAdV5) genome is located between nucleotides 220–400 and consists of seven A-repeats numbered from A1 through A7 (Ostapchuk and Hearing, 2005).

IVa2 is expressed from the early region (E) 2b region located on the minus strand of the viral genome (Binger and Flint, 1984) and contains nuclear as well as nucleolar localization signals (Lutz et al., 1996). Initially, IVa2 was identified as a component of two protein complexes that associate with downstream elements (DEs), DE1 and DE2, of the major late promoter (Lutz and Kedinger, 1996). The homology between sequences of the PD and DE led to the discovery of the role of IVa2 in genome packaging (Zhang and Imperiale, 2000). The binding of IVa2 to the A repeats of the PD is required for the packaging of the viral genome (Zhang and Imperiale, 2000; Zhang et al., 2001; Ostapchuk et al., 2005). IVa2 is also required for recruiting the viral protein L4 22K, an important packaging factor, to the PD (Ostapchuk et al., 2006; Ewing et al., 2007; Tyler et al., 2007). IVa2 is present in equal amounts in all forms of AdV assembly intermediates including the empty capsids, light intermediate particles, young virions and mature virions (Winter and D’Halluin, 1991). Sequence analysis of IVa2 showed the presence of Walker A and Walker B motifs, a characteristic feature of all ATPases. Furthermore, IVa2 has been shown to bind ATP, suggesting that it could function as an ATPase (Ostapchuk and Hearing, 2008). IVa2 mutants carrying mutations in Walker A and Walker B motifs, or lacking a C-terminal fragment encompassing these motifs, resulted in an accumulation of empty capsids implying a defect in genome packaging. Collectively, these results imply that IVa2 is similar to the DNA packaging ATPases observed in dsDNA phages and herpesviruses (Rao and Feiss, 2008; Ostapchuk et al., 2011). It is a common theme in dsDNA phages and herpesviruses that the genome packaging complex or the proteins involved in genome packaging assemble at a unique vertex (Rao and Feiss, 2008). IVa2 has been shown to be located at a unique vertex of mature virions with only 6–8 copies per capsid again suggesting its role as a DNA packaging ATPase (Christensen et al., 2008; Ahi et al., 2013).

The 33K protein of HAdV5 contains 229 amino acid residues and has been identified as a nuclear phosphoprotein found in the infected cell nucleus as a unique granular structure (Gambke and Deppert, 1981). 33K is expressed from the late region 4 (L4) of the major late transcription unit and is a 25 kDa molecular mass, as predicted by its amino acid sequence, but runs anomalously at 35 kDa. 33K is detected in empty capsids but not in mature virions, suggesting that it may be required as a scaffolding protein for capsid assembly (Ahi et al., 2013). A truncated 33K lacking the C-terminal 47 amino acids fails to assemble capsids (Finnen et al., 2001). Similar results were also seen with C-terminal truncation of the 33K protein of bovine adenovirus serotype 3 (BAdV3). Truncation of the BAdV3 33K after the 97th amino acid completely abolished capsid assembly (Kulshreshtha et al., 2004). However, when a stop codon was introduced at 7th or 20th position in the ORFs of 33K in the BAdV3 or HAdV5 genome, respectively, capsid assembly was not affected, but the virus yield was reduced by several fold, indicating that the defect may be at the level of genome packaging (Fessler and Young, 1999; Kulshreshtha et al., 2004). In support of this role as a packaging factor, 33K was shown to interact with IVa2 and localize at a single site on viral capsids (Ahi et al., 2013).

In order to further understand the roles of IVa2 and 33K during genome packaging, we evaluated the oligomeric state of IVa2 and 33K in AdV-infected cells, IVa2 and 33K transfected cells, purified virions or in purified protein preparations and provide the first evidence of ATPase activity of IVa2 in the presence of 33K and the AdV genome.

## Materials and Methods

### Cell Lines, Viruses, and Plasmids

The 293 cell line was derived from the human embryonic kidney cells by transformation with the E1 region of HAdV5 (Graham et al., 1977). The 293cre cell line is derived from 293 cells and constitutively expresses cre recombinase (Parks et al., 1996). 293-IVa2 cells are 293 cells constitutively expressing the HAdV5 IVa2 protein (Ahi et al., 2013). 293-33K cells are generated by stable transfection of 293 cells with pcDNA-33K. These cell lines were maintained in minimum essential medium (MEM) with 10% FetalClone III (Thermo Fisher Scientific Inc., Waltham, MA, USA) and gentamicin (50 μg ml^-1^). Preparation of virus stocks and purification of HAdV5 and ADLC8luc (Parks et al., 1996) was done as described earlier (Graham and Prevec, 1991). Briefly, 293 or 293cre cells were infected, respectively, with HAdV5 or ADLC8luc at a multiplicity of infection (m.o.i.) of 5 plaque-forming units (PFUs) per cell. At 48 h following infection, complete cytopathic effect (CPE) was observed, the infected cells were harvested, and the cell pellet was collected after centrifugation at 3,000 rpm. at 4°C for 10 min. For preparation of the virus stock, the infected cell pellet was re-suspended in PBS^2+^ (Phosphate buffered saline, pH7.4, containing 0.01% MgCl_2_, CaCl_2_) plus 10% glycerol. Viruses were released from infected cells by three cycles of freeze-thaw, and the cell lysates were stored at -80°C. For virus purification, the virus was similarly grown, and the infected cell pellet was processed for virus purification by cesium chloride density-gradient centrifugation (Graham and Prevec, 1991). Virus titers were achieved by plaque assay in BHH2C (bovine-human hybrid clone 2C) cells (van Olphen and Mittal, 2002).

*Escherichia coli* expression plasmids pET28-IVa2 and pET28-33K were created by cloning the synthetic IVa2 or 33K gene cassette (codon-optimized for bacterial expression), respectively, into the pET28a plasmid (EMD Biosciences, San Diego, CA, USA). The pcDNA-IVa2 (Ahi et al., 2013) and pcDNA-33K plasmids were constructed by inserting the synthetic IVa2 or 33K gene cassette (codon- optimized for expression in mammalian cells), respectively, into pcDNA3.1. The 33K gene cassette (either for bacterial or mammalian cell expression) cannot express L4 22K protein (a shorter-version of 33K protein produced in HAdV5-infected cells due to alternative splicing).

### Expression and Purification of 6His-Tagged 33K and IVa2

For expression of 33K, pET28-33K transfected *E. coli* BL21 (DE3) pLysS (EMD Biosciences) cells were grown to an optical density (OD) of 0.8 at 600 nm, and 33K expression was induced for 4 h at 37°C. The purification was done using a His-bind protein purification kit (EMD Biosciences). Purified 33K was dialyzed against 20 mM Tris pH 8.0, 400 mM NaCl, and 10% glycerol. Protein concentration was estimated by a Coomassie protein assay (Thermo Scientific, Rockford, IL, USA). Final yield of 33K was ∼3 mg from 350 ml culture.

For expression of IVa2, *E. coli* BL21 (DE3) pLysS (EMD Biosciences) cells containing pET28-IVa2 were grown to an approximate 1.6 OD at 600 nm, and the expression was induced for 16 h at 12°C. Purification was done by immobilized metal ion affinity chromatography with an ÄKTA Prime Plus System (GE Healthcare, Piscataway, NJ, USA) at 4°C using a HiTrap NiNTA column (GE Healthcare). IVa2 containing fractions were pooled and further purified at 4°C using a HiPrep 16/10 SP-XL column (GE Healthcare). After the previous step was completed, IVa2 containing fractions were again pooled, dialyzed against buffer containing 50 mM Tris, 500 mM NaCl, 5% glycerol, concentrated to 1 mg ml^-1^ using 30 kDa cutoff Amicon ultra-15 centrifugal filter units (Millipore, Billeria, MA, USA), and stored at -80°C in aliquots. The final yield of IVa2 was ∼5 mg from a 1000 ml culture.

### SDS–PAGE Under Reducing and non-Reducing Conditions

Purified proteins, purified virus, or cell extracts were treated with reducing Laemmli buffer (50 mM Tris, pH 6.8, 10% glycerol, 2% SDS, 5% beta-mercaptoethanol, and 0.02% bromophenol blue) followed by boiling for 5 min, or non-reducing Laemmli buffer (50 mM Tris, pH 6.8, 10% glycerol, 2% SDS, and 0.02% bromophenol blue) and separated on SDS-PAGE. The gel was either stained with Coomassie blue or processed for immunoblotting. The immunoblotting was performed using polyclonal anti-IVa2 and anti-33K antibodies raised in rabbits against bacterially purified IVa2 or 33K as described earlier (Ahi et al., 2013). 33K shares its N-terminal 105 amino acids residues with another HAdV5 protein, L4 22K, but our anti-33K antibody did not react with L4 22K, which runs at ∼30K (Oosterom-Dragon and Anderson, 1983; Ewing et al., 2007). Our anti-33K antibody detected only a single band at ∼35K, suggesting that it did not react with L4 22K.

### Transmission Electron Microscopy

Glow-discharged 400 mesh copper with carbon-coated formvar grids (Electron Microscopy Sciences, Hatfield, PA, USA) were used for transmission electron microscopy (TEM) of purified protein samples. The grids were floated on protein sample droplets for 1 min, rinsed with 20 mM Tris, pH 7.5 to remove unbound proteins, and stained with 2% uranyl acetate. Imaging was done with FEI/Philips CM-100 TEM (Philips/FEI Corporation, Eindhoven, Netherlands). Images were captured at 92,000× and 180,000×.

### Purification of the Terminal Protein-Associated HAdV5 Genome

The terminal protein (Tp)-associated HAdV5 genome (Tp-DNA) was isolated from purified HAdV5 as described (Graham and Prevec, 1991). Briefly, purified virus in TE buffer (10 mM Tris and 1 mM EDTA, pH 8.0) was mixed with an equal volume of 8 M guanidine hydrochloride in TE buffer and incubated on ice for 30 min, mixing every 10 min. Cesium chloride and guanidine chloride concentrations in the mixture was adjusted to 2.8 M and 4 M, respectively. The solution was centrifuged at 55,000 rpm in the VTi65.1 rotor (Beckman Coulter, Indianapolis, IN, USA) at 4°C for 16 h. Fractions of 0.5 ml size were collected and analyzed for the presence of Tp-DNA by agarose gel electrophoresis. Fractions containing Tp-DNA were pooled and dialyzed against TE buffer. The Tp-DNA concentration was determined spectrophotometrically.

### ATPase Assay

The assay for determining ATPase activity of IVa2 was done in the presence or absence of purified 33K and Tp-DNA. The purified IVa2 in the absence or presence of equal amounts of 33K was dialyzed against buffer containing 50 mM Tris, pH 7.5, 400 mM NaCl, and 10% glycerol. ATPase reactions contained 50 mM Tris, pH7.5, 100 mM NaCl, 10 mM MgCl_2_, 0.5 mM ATP, 1 μg protein/s (IVa2+33K, or IVa2, or 33K) with or without 5 μg Tp-DNA in a 200 μl volume. Reactions were incubated at 37°C for 30 min. At end of the incubation, the inorganic phosphate concentration in the reactions was measured using ATPase Assay System (Innova Biosciences, Babraham, Cambridge, UK).

## Results

### Oligomeric State of IVa2 and 33K in Virus-Infected Cells, Purified Virus Preparations and Transfected Cells

**FIGURE 1:**
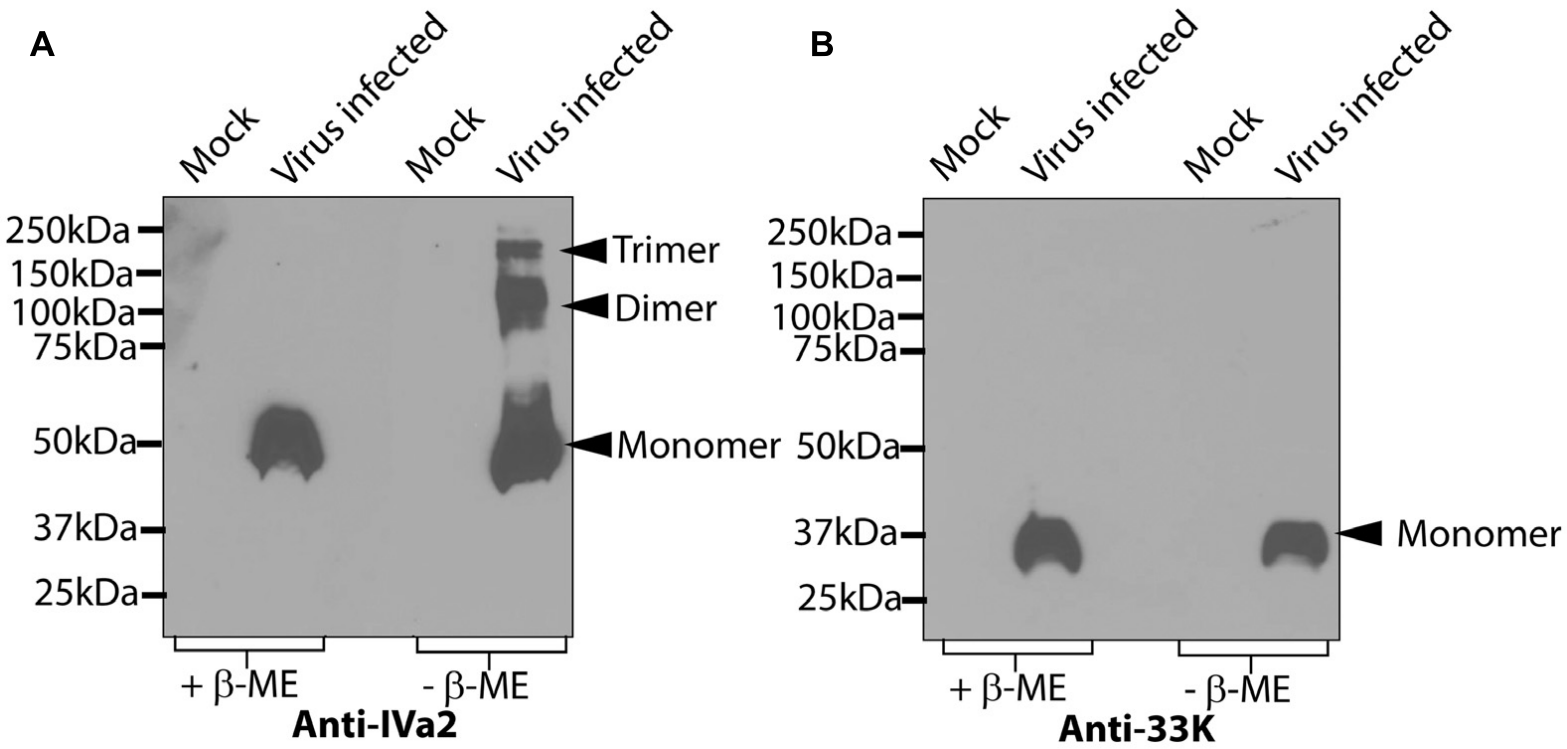
**Oligomeric state of IVa2 and 33K proteins in virus infected cells.** Mock or HAdV5 infected cell lysates were prepared at 36 h post-infection and treated in reducing or non-reducing conditions. Twenty microgram cell lysates were separated on SDS-PAGE, the proteins transferred to membrane and analyzed by Western blots with anti-IVa2 **(A)** or anti-33K **(B)** antibodies. Positions of monomer, dimer, and trimer of IVa2 are indicated. Molecular mass markers are indicated on the left.+β-ME, beta-mercaptoethanol was used as a reducing agent.

It has been shown that both IVa2 and 33K are located at a unique vertex of the viral capsids (Christensen et al., 2008; Ahi et al., 2013). We therefore proposed that IVa2 and 33K might assemble into ring-like oligomers with a central channel large enough to allow the passage of the viral genome. To this end, we analyzed the oligomers of IVa2 and 33K in virus-infected cells. Lysates of HAdV5-infected cells were processed for SDS–PAGE in both reducing and non-reducing conditions, followed by a Western blot with anti-IVa2 or anti-33K antibody. In reducing conditions, IVa2 migrated at a molecular mass of 50 kDa as expected for a monomer (**Figure 1A**). However, in non-reducing conditions, IVa2 was detected at the molecular mass expected for monomers, dimers and higher order oligomers (**Figure 1A**). The 33K was detected as a monomer in both the reducing and non-reducing conditions (**Figure 1B**).

**FIGURE 2:**
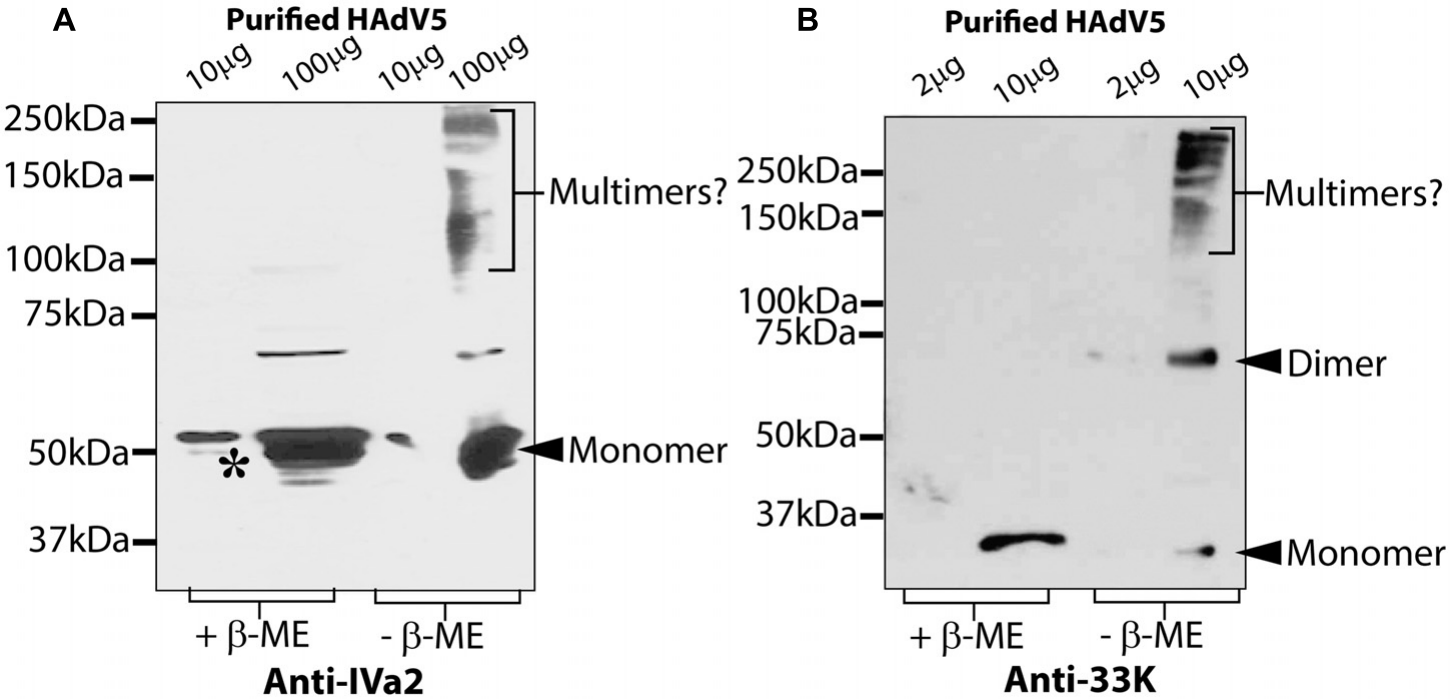
**Oligomeric state of IVa2 and 33K proteins in purified virus.** Indicated amounts of purified HAdV5 virus were treated in reducing or non-reducing conditions and analyzed by Western blots with anti-IVa2 **(A)** or anti-33K **(B)** antibodies. Positions of monomer and multimers of each protein are indicated. Molecular mass markers are indicated on the left. +β-ME, beta-mercaptoethanol was used as a reducing agent.

Next, we analyzed the oligomers of IVa2 and 33K in purified virus particles. Purified HAdV5 virions were analyzed by SDS-PAGE in both reducing and non-reducing conditions and Western blots with anti-IVa2 or anti-33K antibody. In reducing conditions, the anti-IVa2 antibody reacted with two bands running close together with a proportionally lesser amount of the smaller band indicated by an asterisk (**Figure 2A** – lane with 10 μg protein in reducing condition). The smaller band may represent an unmodified form of IVa2. Under non-reducing conditions, a smear-like pattern was detected above 100 kDa, possibly reflecting dimers and multimers of IVa2 or its association with other capsid proteins. The anti-33K antibody reacted with a single band in reducing conditions, whereas in non-reducing conditions, a clear band was detected at ∼70 kDa, the expected position for a dimer (**Figure 2B**). In addition, a smear-like pattern appeared above 150 kDa reflecting higher oligomers of 33K or its association with other capsid proteins. A small fraction of 33K was also detected at the monomer position.

**FIGURE 3:**
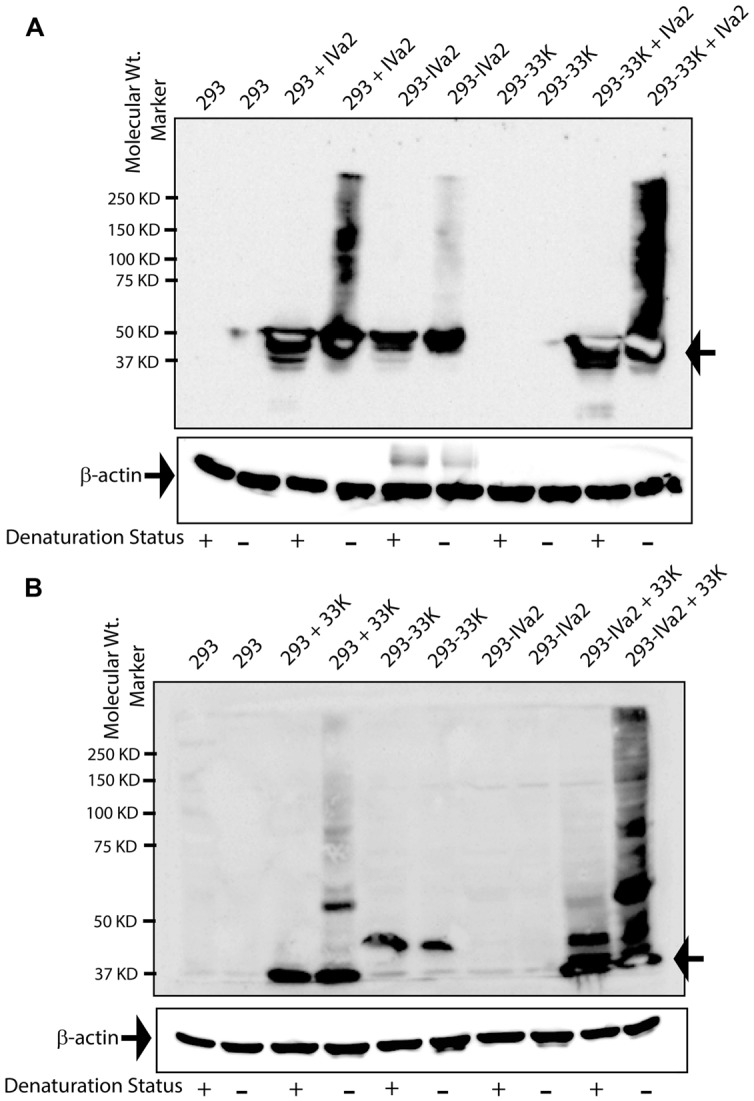
**Oligomeric state of IVa2 and 33K proteins in transfected cells. (A)** Mock or pcDNA-IVa2 transfected 293 or 293-33K cells were harvested at 36 h post-transfection and treated with (+) or without (-) beta-mercaptoethanol (β-ME). 293-IVa2 cells were used as controls. **(B)** Mock or pcDNA-33K transfected 293 or 293-IVa2 cells were harvested at 36 h post-transfection and treated in reducing or non-reducing conditions. 293-33K cells were used as controls. Cell lysates were analyzed by Western blots with anti-IVa2 **(A)** or anti-33K **(B)** antibody. Similar results were obtained in four independent transfection studies. For monitoring equal loading, the Western blots for beta-actin were conducted and shown as an insert at the bottom of each blot. Positions of monomer and higher order multimers are indicated. Molecular mass markers are indicated on the left. In the figure, 293 cells transfected with pcDNA-IVa2 are denoted as 293 + IVa2; 293 cells stably expressing IVa2 are denoted as 293-IVa2; 293 cells transfected with pcDNA-33K are denoted as 293 + 33K; 293 cells stably expressing 33K are denoted as 293-33K.

We next sought to evaluate the IVa2 and 33K oligomers in the absence of other viral factors to ascertain whether the presence of 33K influences the oligomer formation by IVa2 and vice versa. These experiments involved transient transfections in 293, 293-IVa2, and 293-33K cells. To study IVa2 oligomers in both the absence and presence of 33K, the 293, or 293-33K cells were transfected with pcDNA-IVa2 and processed for Western blot with anti-IVa2 or anti-33K antibody. IVa2 oligomers were detected in the 293 cells transfected with pcDNA-IVa2 (293 + IVa2) in non-reducing conditions (**Figure 3A**). A far greater amount of IVa2 oligomers was detected when 33K was also present (compare 293 + IVa2 and 293-33K + IVa2), suggesting that 33K promotes oligomer formation by IVa2. Similarly, much more 33K oligomers were detected when IVa2 was also present (compare 293 + 33K and 293-IVa2 + 33K), suggesting that IVa2 also promotes oligomer formation by 33K (**Figure 3B**). It is noteworthy that both IVa2 and 33K oligomers do not seem to form in significant amounts when expressed individually in their respective stable cell lines. This could be due to a lower amount of protein being expressed in stable cell lines (293-IVa2 and 293-33K) as compared to transient transfections (293 + IVa2 and 293 + 33K). However, the detected level is sufficient to promote the oligomer formation of its ‘interacting partner’ expressed at a higher level in transient transfection.

### Expression and Purification of IVa2 and 33K

**FIGURE 4:**
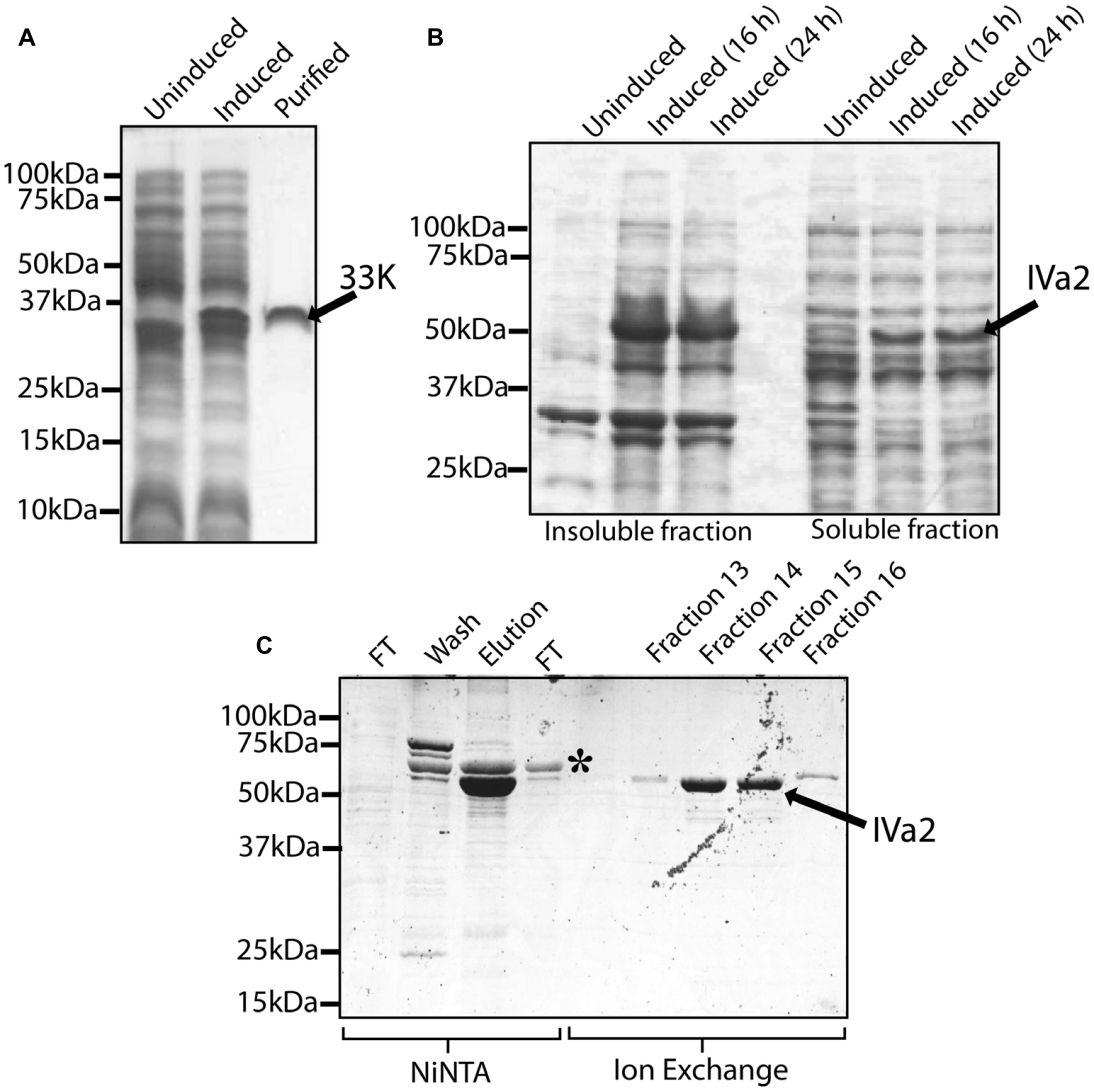
**Expression and purification of 33K and IVa2. (A)** Expression and purification of 33K: whole cell extracts of un-induced culture, induced culture and 5 μg purified protein were processed for SDS-PAGE and the gel was stained with Coomassie blue. **(B)** Expression of IVa2 in soluble form: insoluble and soluble fractions of un-induced cultures or cultures induced for 16 or 24 h at 12°C were separated on SDS-PAGE gel, and the gel was stained with Coomassie blue. **(C)** Purification of IVa2 expressed in the soluble fraction: IVa2 was purified, first by NiNTA affinity chromatography, and then by Ion exchange chromatography. Samples from various stages of purifications were collected and analyzed SDS-PAGE gel and Coomassie blue staining. FT, flow through.

In order to characterize the oligomeric state of IVa2 and 33K in the absence of other proteins, IVa2 and 33K were expressed in bacteria and purified. Purified 33K (**Figure 4A**) migrated as a single band at the molecular mass of approximately 35 kDa. Earlier studies had reported a similar migration pattern of 33K (Oosterom-Dragon and Anderson, 1983).

The IVa2 was found in both soluble and insoluble fractions (**Figure 4B**). The purified preparation contained a major species, indicated by an arrow, migrating at a molecular mass of 53 kDa (**Figure 4C**) as expected for IVa2. The identity of this band was confirmed by Western blot (data not shown). In addition, an impurity (asterisk) migrating at ∼60 kDa was also found that did not react with anti-IVa2 antibody (data not shown). The impurity was removed using a HiPrep 16/10 Sp XL cation exchange column (**Figure 4C**). Identities of the purified IVa2 and 33K proteins were confirmed by mass spectrometric analysis (data not shown).

### Oligomeric State of Purified IVa2 and 33K Proteins

**FIGURE 5:**
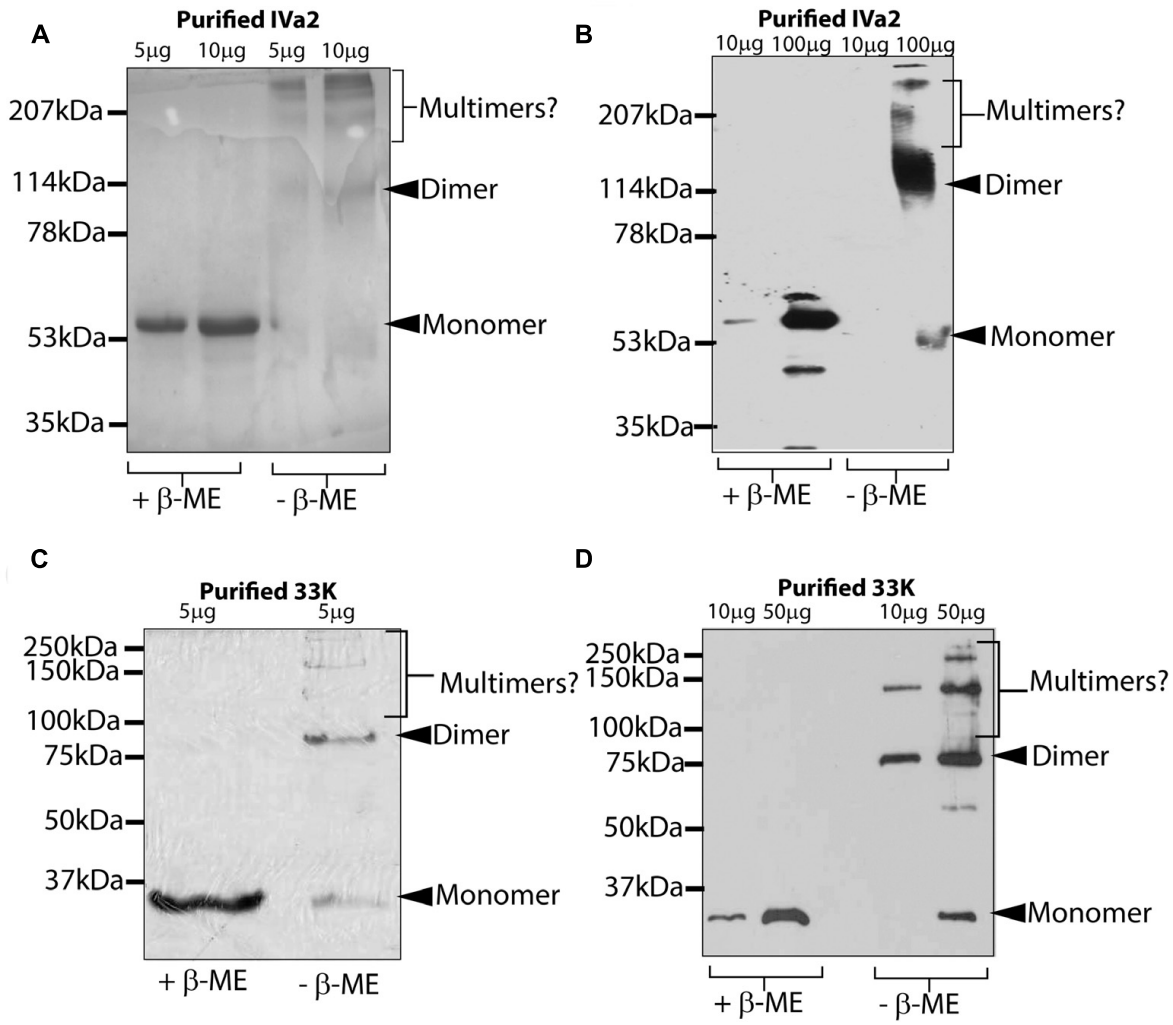
**Oligomeric state of purified IVa2 and 33K proteins.** Indicated amounts of purified IVa2 **(A,B)** or 33K **(C,D)** proteins were treated in reducing or non-reducing conditions and separated on SDS-PAGE gel. Gel was either processed for Coomassie blue staining **(A,C)** or processed for Western blots with anti-IVa2 **(B)** or anti-33K **(D)** antibodies. Positions of monomer, dimer, or higher multimers of each protein are indicated.

The purified IVa2 and 33K proteins were subjected to SDS–PAGE in both reducing and non-reducing conditions followed by Coomassie staining or Western blot. In reducing conditions, both IVa2 (**Figures 5A,B**) and 33K (**Figures 5C,D**) migrated as monomers at the molecular mass of approximately 53 and 35 kDa, respectively. In addition to the full-length IVa2 band, a faster migrating band was detected by anti-IVa2 antibody, suggesting that it may be a degradation product of IVa2 (**Figure 5B**). In non-reducing conditions, IVa2 appeared as a series of bands corresponding to the molecular mass of dimers and higher order multimers. Similar results were obtained by Western blot with an anti-IVa2. Similarly, in non-reducing conditions, 33K was found in dimers and higher order multimers, but a significant fraction was also present as a monomer (**Figure 5C**). Similar results were obtained by Western blot with anti-33K antibody (**Figure 5D**).

**FIGURE 6:**
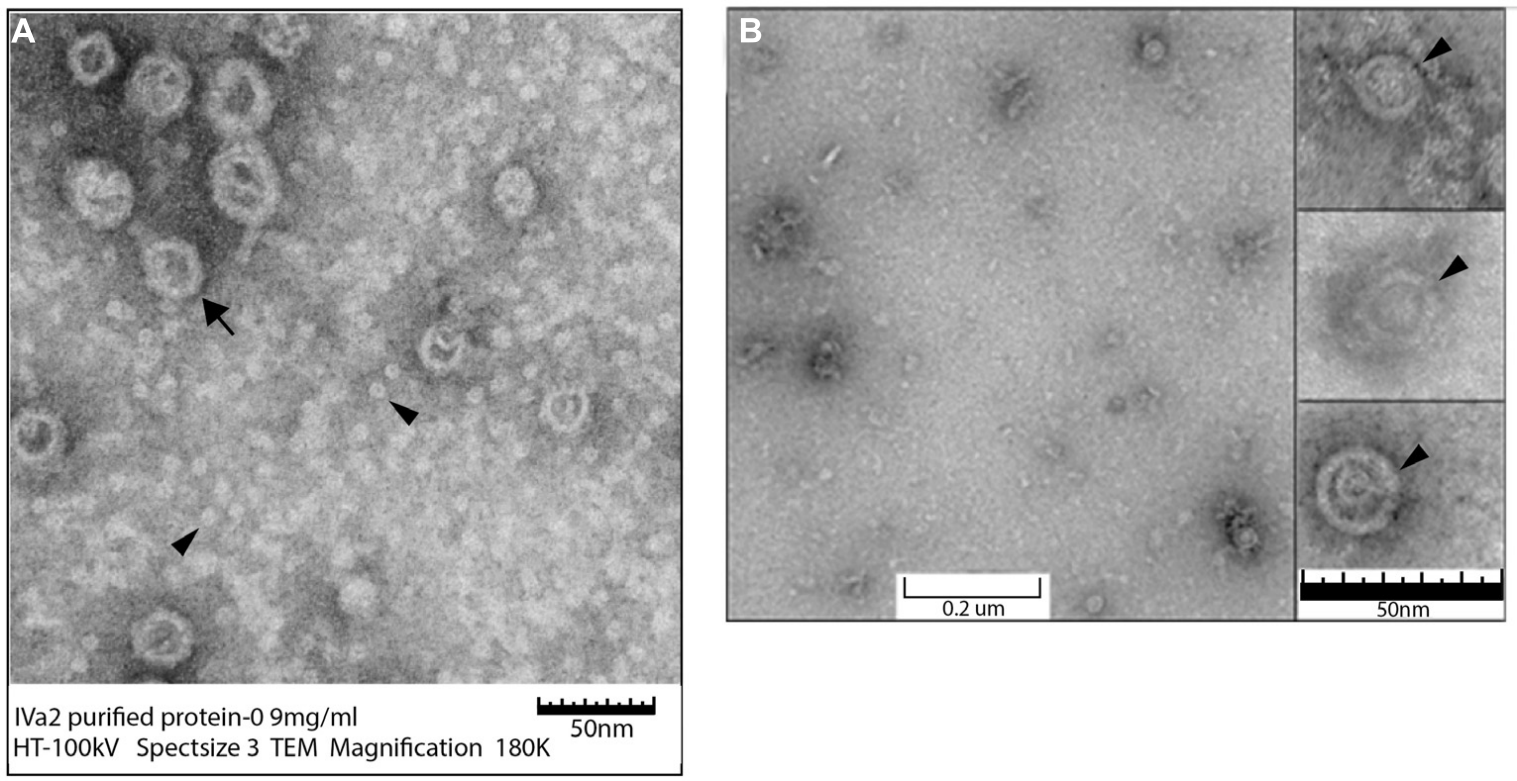
**Transmission electron microscopy (TEM) of purified IVa2 and 33K proteins.** Purified proteins were applied to grids and imaged with TEM. **(A)** IVa2 (180,000×). **(B)** 33K (90,000×). Inset to **(B)**: individual 33K particles at 250K magnification.

In order to determine the structural features of these oligomers, the purified proteins were analyzed by TEM. IVa2 was found to be distributed in two populations: large rings (**Figure 6A**; arrow) and smaller structure (**Figure 6A**; arrow head) populations (**Figure 6A**). The smaller structures were roughly of 7–9 nm in diameter. The larger rings were more variable in size ranging from 30 to 50 nm in diameter. Purified 33K was found in rings of approximately 15–17 nm in diameter (**Figure 6B**) forming a cone-like structure (Inset, **Figure 6B**; arrowhead).

### IVa2 and 33K Associate with HAdV5 Packaging Domain

The association of IVa2 with the AdV PD (Ostapchuk et al., 2005) and with 33K (Ahi et al., 2013) during virus infection has already been demonstrated. To determine whether 33K associates with the AdV PD in virus-infected cells, a DNA-protein pull down assay was performed using a 5^′^biotin-labeled AdV PD and virus-infected cell extract as described earlier (Wu, 2006). The HAdV5 PD sequence, between nucleotides 191–360, was labeled with biotin at both ends using 5^′^ biotin-labeled forward and reverse primers for PCR amplification.

**FIGURE 7:**
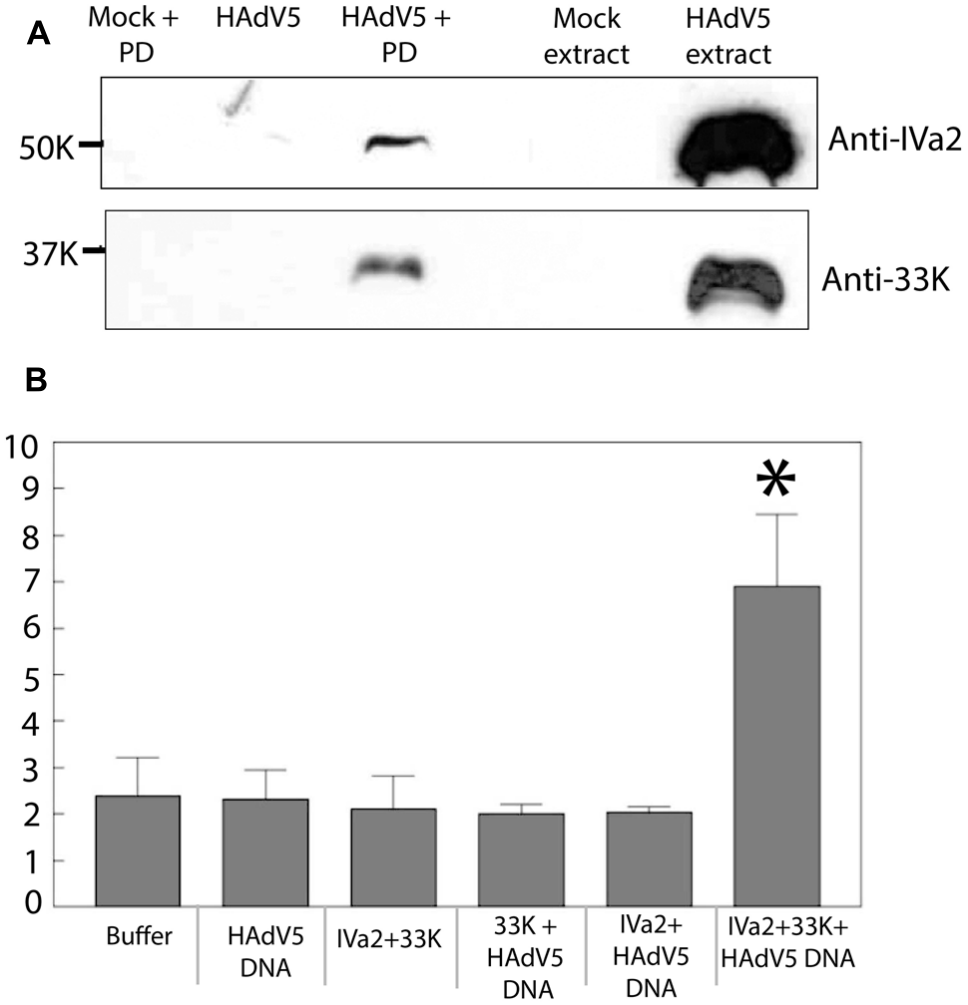
**(A)** Binding of IVa2 and 33K to the packaging domain (PD): Nuclear extracts of ADLC8cluc infected 293cre cells were treated with 5^′^-biotin labeled HAdV5 packaging signal sequences and the DNA protein complexes were purified using streptavidin agarose beads. Purified proteins were analyzed by Western blots with anti-IVa2 or anti-33K antibodies. Positions of IVa2 and 33K bands are indicated. **(B)** ATPase activity of IVa2 in the presence of 33K and the HAdV5 genome: ATPase assay was performed with purified IVa2 in the presence of 33K, or HAdV5 Tp-DNA or both. Concentration of inorganic phosphate (Pi), indicated on the *Y*-axis, in reactions at end of reaction incubation is indicated on y axis. *P < 0.05. The error bar represents SD.

The rationale of using ADLC8cluc-infected 293cre cells was based on the fact that the PD of ADLC8cluc is flanked by loxP sites and therefore, will be removed by the loxP-cre recombinase system and, thus, ensure an abundance of free IVa2 and 33K for an *in vitro* binding assay. Nuclear extracts of ADLC8cluc-infected 293cre cells were incubated with a 5^′^biotin-labeled AdV PD, followed by purification of DNA-protein complexes using streptavidin agarose beads. The purified complexes were analyzed by Western blots using anti-IVa2 or anti-33K antibody. Both IVa2 and 33K were present in the purified complexes with the biotin-labeled PD (**Figure 7A**), but were not found in the absence of a 5^′^biotin-labeled AdV PD or in the presence of a non-specific DNA (not shown). This result indicates that IVa2 and 33K interact with the AdV PD *in vitro*.

### 33K Stimulates ATPase Activity of IVa2 in the Presence of Viral Genome

IVa2 is thought to act as an ATPase during genome packaging; however, its ATPase activity has not been demonstrated yet. Given that IVa2 interacts with 33K, we hypothesized that 33K is required for activation of ATPase activity of IVa2. In order to test this, purified IVa2 was dialyzed in both the absence and presence of purified 33K against 33K dialysis buffer (50 mM Tris pH 7.5, 400 mM NaCl, and 10% glycerol) with an intention to form complexes between the two proteins. Protein concentration of the resulting mixture was determined by a Coomassie protein assay. The dialyzed mixture was incubated in an ATPase reaction mixture in the presence or absence of Tp-DNA (HAdV5 genome complexed with TPs). Reactions were incubated at 37°C for 30 min, and the concentration of inorganic phosphate in each reaction was measured using the Innova Biosciences ATPase assay system. A statistically significant (*P* < 0.05) increase in the Pi concentration was observed in reactions containing all three components – IVa2, 33K, and the Tp-DNA. However, no significant increase was observed in reactions that contained only IVa2 + 33K, 33K + Tp-DNA, or IVa2 + Tp-DNA (**Figure 7B**). Given that the Pi concentration in reactions when any one of the three components was omitted was similar to that of buffer alone and that there was only a threefold increase in Pi concentration when all three components were present suggests that other components are required for maximal ATPase activity. Nevertheless, this result does suggest that 33K stimulates ATPase activity of IVa2 in the presence of viral genome.

## Discussion

Adenoviruses are presumed to package their genome into pre-assembled empty capsids via a mechanism similar to bacteriophages and herpesviruses. AdV assembly continues along a pathway that proceeds through the assembly of the hexons and pentons, the assembly of empty capsids, and the specific recognition of the PD followed by packaging of the viral genome by the packaging machinery into empty capsids. This is followed by the proteolytic cleavage of precursor proteins by a viral protease leading to the final maturation of viral particles. IVa2 is believed to function as an equivalent to the DNA packaging ATPases in bacteriophages and herpesviruses. The 33K protein also has been reported to be essential for capsid assembly and genome packaging (Wu et al., 2013). In an attempt to further characterize the roles of IVa2 and 33K in capsid assembly and genome packaging, we analyzed the oligomer status of IVa2 and 33K in virus-infected cells, purified virus, IVa2, and 33K transfected cells and bacterial-expressed proteins. We showed that both IVa2 and 33K form oligomers and enhance oligomer formation by each other to provide the first evidence of the ATPase activity of IVa2.

Disulfide linkages have been shown to be necessary for the formation of the portal ring by the portal protein UL6 and also for the assembly of procapsids compatible with HSV-1 packaging (Albright et al., 2011). In our experiments, detection of slower migrating bands in non-reducing conditions in virus-infected cells and purified virus extracts with anti-IVa2 antibody suggest that IVa2 forms oligomers and that the oligomers are stabilized by disulfide linkages. Since the cell lysates and purified virus were treated with Laemmli buffer containing 2% SDS, it is unlikely that the observed oligomers are due to non-covalent interactions. The presence of a substantial amount of IVa2 as a monomer in the virus-infected cells and purified virus extracts in non-reducing conditions indicates that not all IVa2 subunits are stabilized by the disulfide linkages.

Similar to IVa2, 33K also appeared to form dimer and higher oligomers in the purified virus extract. Surprisingly, the dimer and higher oligomers of 33K were not observed in virus-infected cells. Since 33K is a multifunctional protein, involved in late protein transcription, capsid assembly as well as genome packaging (Ali et al., 2007; Wu et al., 2013), it is likely that, in virus-infected cells, it remains as a monomer before being associated with virions. It is also possible that the relative amount of virion-associated 33K in virus-infected cells is very low compared to its free fraction. Oligomers, along with some monomer, were also observed in purified IVa2 and 33K in non-reducing conditions (**Figure 5**). In addition, oligomers of both IVa2 and 33K were observed in transfected cells. The presence of IVa2 and 33K seems to drastically enhance oligomer formation by 33K and IVa2, respectively, suggesting a functional interaction of these proteins.

TEM analysis of purified IVa2 did not reveal large aggregates even at a high concentration of 2 mg ml^-1^. This suggests that the protein is soluble, and oligomers detected by SDS–PAGE are not due to improper folding or non-specific aggregation. The structures of IVa2 observed by TEM can be classified into two types: small structures with a diameter of ∼7–9 nm and larger “rings” with a diameter between 30 and 50 nm. The diameter of a globular protein of 50 kDa is expected to be 4–5 nm (Kiselyova and Yaminsky, 2004), suggesting that the small structures may be dimers. IVa2 was shown to be present at a unique vertex and has only 6–8 copies per capsid (Christensen et al., 2008). It is likely that the basic unit of the IVa2 oligomer at a unique vertex is a dimer similar to the smaller structures observed by TEM. Large terminases of all dsDNA phages including T4, T7, SPP1, and P22 (Nemecek et al., 2007; Rao and Feiss, 2008) are known to exist as monomers in solution, but, in packaging, the ATPase gp16 of ph29 forms trimers and ring-like oligomers with a ∼9.5 nm diameter at concentrations between 0.2 and 0.8 mg ml^-1^ (Koti et al., 2008). Our preliminary TEM studies of purified 33K indicate that it organizes into ring-like structures with a diameter of ∼17 nm. Further analyses of IVa2 and 33K at a higher resolution will be required to determine their structural features.

It appears that IVa2 serves as the packaging ATPase. The absence of 33K in mature AdV (Ahi et al., 2013) indicates that 33K does not serve as a portal. In addition to the portal and packaging ATPase, the third component of the packaging motor is a small terminase which also forms ring-like oligomers. Analysis of the oligomeric state of the purified small terminase gp16 of T4 and related phages which yield a ladder of bands suggest the existence of these proteins as larger oligomers (Leffers and Rao, 2000; Koti et al., 2008; Gao and Rao, 2011). Purified gp16 is shown to form rings of ∼8 nm diameter with a central hole of ∼2 nm (Lin et al., 1997). The ring-like oligomers of the small terminase of a T4-like phage assemble with 8–12 subunits and appear to have a cone-like structure (Sun et al., 2012). The small terminase gp3 of phage P22 forms a ring-like structure of ∼9 nm in diameter with a central hole and outwardly projecting spikes (Nemecek et al., 2007). In the case of phage SPP1, the two or three ring-shaped decamers of g1p interact with g2p and assemble at a unique vertex (Camacho et al., 2003).

The small terminase protein enhances the ATPase activity of the larger terminase which, by itself, is a weak ATPase (Leffers and Rao, 2000; Camacho et al., 2003; Rao and Feiss, 2008). IVa2 is thought to be the packaging ATPase of AdV equivalent to the large terminase of phage. It should be noted that IVa2 does not have terminase activity as such activity is not needed for AdV replication. We recently demonstrated that 33K interacts with IVa2 and extensively co-localizes in the absence of other viral proteins (Ahi et al., 2013). Additionally, our results suggest that IVa2 and 33K promote oligomer formation by each other implying interaction with 33K may be required for ATPase activity of IVa2. Along the same line, the ATPase activity of IVa2 was detected in the presence of 33K and the viral genome. However, only a low level of ATPase activity was detected which may be partly due to several factors: improper folding, lack of post-translation modifications of IVa2 and 33K when expressed in bacteria, or the absence of other important components. The characterization of the ATPase function of IVa2 is very preliminary at this stage, but it certainly has provided a platform for future experiments to determine the optimal conditions for ATPase activity. Additionally, the necessity of other components such as a portal, other interacting partner/s, or the procapsid should be systematically examined to determine the optimum conditions for ATPase function of IVa2.

**FIGURE 8:**
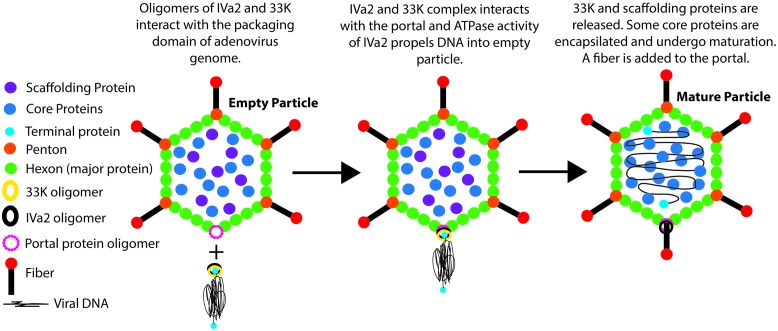
**Diagrammatic representation of the role of IVa2 and 33K in AdV genome packaging.** Empty virions are assembled from the major capsid proteins with the help of the portal protein (unknown), scaffolding protein (unknown), and core proteins. The AdV genome is recognized by the packaging proteins IVa2 and 33K (other packaging proteins are not shown for the sake of simplicity) due to the presence of the PD near the left end of the genome. In association with 33K, the ATPase activity of IVa2 propels the viral genome into an empty virion particle through the portal. It is anticipated that the portal protein will interact with IVa2. Upon completion of packaging, the 33K protein and scaffolding proteins are released. Some core proteins may encapsidate during and/or after the genome packaging and undergo maturation. Finally, a fiber molecule is assembled at the portal vertex.

Several lines of evidence indicate that 33K is the small terminase for AdV. 33K is present in empty but not in mature capsids, is located at a unique site on the capsid, and interacts with the large terminase equivalent of IVa2 (Ahi et al., 2013). In addition, the results presented in this manuscript elucidate the formation of ring-like oligomers of 33K and its stimulation of IVa2 ATPase activity. The role of IVa2 and 33K in the AdV packaging is depicted in a diagrammatic form (**Figure 8**). A BAdV3 mutant carrying a stop codon at position 7 in the ORF of the 33K showed significant reduction in mature particles compared to the wild type BAdV3, suggesting a defect in the process of genome packaging (Kulshreshtha et al., 2004). Similarly, introduction of a stop codon at position 20 in the ORF of 33K in HAdV5 decreased virus yield (Fessler and Young, 1999). These observations suggest that, analogous to the small terminase protein, the 33K plays an important role in genome packaging. Another mutant of BAdV3 carrying a stop codon at position 97 in the ORF of the 33K failed to assemble virus particles, pointing toward a role of 33K in capsid assembly (Kulshreshtha et al., 2004). Along the same line of thought, deletion of the C-terminal 47 amino acids of 33K of HAdV5 completely abolished capsid assembly (Finnen et al., 2001). Taken together, these results imply that a domain in the C-terminus of 33K is required for capsid assembly, whereas, the function of 33K in genome packaging is located in its N-terminus. Thus, the 33K seems to play important roles in both capsid assembly and genome packaging.

## Conflict of Interest Statement

The authors declare that the research was conducted in the absence of any commercial or financial relationships that could be construed as a potential conflict of interest.
